# Effectiveness of a Mindfulness-Based Group Intervention for Chinese University Students with Sleep Problems

**DOI:** 10.3390/ijerph19020755

**Published:** 2022-01-10

**Authors:** Lin Fu, Shuang Wei, Jin Cheng, Xueqi Wang, Yueyue Zhou, Yi Li, Hao Zheng

**Affiliations:** 1 Faculty of Humanities and Social Sciences, Beijing University of Technology, Beijing 100124, China; linfu@bjut.edu.cn (L.F.); wojiaowangxueqi@163.com (X.W.); liyi_602@163.com (Y.L.); 2 Beijing Institute of Education, Beijing 100097, China; chengjin_18@outlook.com; 3 School of Psychology, Henan University, Kaifeng 475004, China; zhouyueyue@henu.edu.cn; 4 Department of Psychology, University of Alberta, Edmonton, AB T6G 2E9, Canada; hzheng16@ualberta.ca

**Keywords:** group-based, mindfulness, mindfulness-based intervention, sleep problems, university students

## Abstract

The increasing prevalence of sleep disorders among university students should be taken seriously. Group counseling involving a mindfulness-based strategy may help prevent students from developing insomnia and subsequent mental health disorders. This study aimed to evaluate the ameliorating effects of a mindfulness-based group intervention on sleep problems and emotional symptoms in university students in China. Twenty-one university students (16 females, 22.71 ± 4.28 years) who were not on medication were recruited and assigned to the intervention group based on the criterion of high levels of sleep problems. Additionally, twenty-four university students (19 females, 24.50 ± 0.93 years) were included as a nonrandomized control group. Individuals in the intervention group participated in a two-hour group intervention once a week for eight sessions. All participants completed self-reported questionnaire baseline tests, postintervention tests, and one-month follow-ups on mindfulness, sleep quality, anxiety and depressive symptoms. Repeated-measures ANOVA was performed. The results revealed significant intervention effects, with significant differences observed between the two groups in mindfulness and sleep quality. However, there was no significant effect of the intervention on anxiety and depressive symptoms. This study contributes to a better understanding of the effectiveness of mindfulness-based intervention in addressing sleep problems in university students.

## 1. Introduction

University students are a high-risk population for developing sleeping problems, mainly because they have to cope with multiple stressors, including leaving home, increased independence, new peer groups, and changes in social situations [1]. Poor chronic sleep contributes to insomnia, which impacts the future health and functioning of youths [2]. Sleeping problems may cause university students to suffer from daytime dysfunctions, such as poor academic performance [3], and even behavioral problems such as substance abuse [4]. It is common for university students with insomnia to suffer from mental health issues, such as chronic fatigue, anxiety, stress, and even depression [5]. A survey showed that 18.7% to 21.4% of Chinese college students have sleep problems [6]. Therefore, there is a need to find an effective way of improving students’ sleep.

Current popular practices for sleep improvement are pharmacotherapy and/or psychotherapy. Pharmacotherapy is extensively used in clinical care as it has significant efficacy in reducing insomnia symptoms, especially among patients with psychological problems [7,8]. However, pharmacotherapy tends not to be the first-line treatment for sleep problems for college students due to potential side effects and uncertain long-term effects [9]. Psychotherapies, such as cognitive-behavioral therapy, have shown moderate effects in enhancing sleep quality among university students [10]; however, such approaches tend to require a qualified psychiatrist or psychologist with supervised clinical training, which limits their extensive use within universities where the prevalence of insomnia is high and qualified therapists are insufficient.

Mindfulness is an experiential process during which people’s attention is directed purposefully and consciously to what is happening in the present moment. A person may focus on the present moment and on the surrounding environment and activities in a nonjudgmental manner in a mindful state [11]. Multiple studies have provided empirical evidence suggesting that the level of mindfulness was significantly associated with a series of psychological outcomes, including the reduction of depressive symptoms, anxiety, stress [12,13], and the improvement of sleep quality [14]. Furthermore, mindfulness training was confirmed to be effective for increasing sleep quality and buffering distress [15,16].

Mindfulness-based intervention (MBI) is a relatively emerging psychotherapy intervention that enhances an individual’s mindfulness, including both formal (e.g., sitting meditate on, body scanning, and Metta meditation) and informal techniques (e.g., mindfulness eating, mindfulness walking), and has been found to be an effective treatment for university students [17,18]. MBIs emphasize the nonjudgmental focus on and awareness of the present moment, emotional and behavioral self-regulation, and mindfulness meditation practice [19], and they are appropriate for university students because they are easily learned and can be practiced conveniently in daily life, which increases resilience to stress [20]. Additionally, MBI with groups has become increasingly popular within universities, since researchers have confirmed that group-based interventions have significant influence on the alleviation of psychological symptoms [21,22].

MBI provides participants an opportunity to cultivate nonjudgmental awareness and concentrate on the present moment by practicing mindfulness, a calming state which may decrease pre-sleep arousal and worry and improve sleep [23,24,25]. There are multiple empirical studies of MBI demonstrating that mindfulness-related practice can be effective in the treatment of sleep problems by significantly reducing the severity of insomnia and sleep latency and can prolong total sleep time and improve sleep efficiency and perceived sleep quality [8,15,26,27,28]. A pilot study showed that a mindfulness-based, in-school group intervention could help adolescent girls to improve their sleep quality [29]. Furthermore, a meta-analysis study revealed that MBI could ameliorate sleep problems and fatigue among breast cancer patients [30,31]. However, contradictory findings emerged in a meta-analysis study regarding MBIs in university students, with MBIs shown to improve anxiety, depression, and mindfulness with small to moderate effect sizes but to yield no benefit with respect to sleep [17]. Long-term benefits were not seen in MBI for insomnia [32] Accordingly, it remains to be investigated whether mindfulness-based training could alleviate university students’ sleep problems.

The purpose of this study was to examine the effectiveness of group-based MBI in preventing sleep problems among university students in China. Since group-oriented MBI has not been studied in university students, we first examined the feasibility and effectiveness of an eight-session, face-to-face group intervention for reducing sleep problems in individuals in a university setting. We expected the intervention, through mindfulness practices including formal and informal techniques, to enhance participants’ awareness of the present moment. In addition, given the close associations between mental health outcomes and sleep quality, we hypothesized that group-based MBI could be effective for reducing sleep-related emotional problems, including anxiety and depressive symptoms, among university students with sleep problems.

## 2. Materials and Methods

### 2.1. Design

This study used a quasi-experimental design to compare the extent to which the control and intervention conditions affected sleep quality of participants pretreatment, posttreatment, and four weeks after the treatment. The intervention group was recruited online within the university where the researchers worked from a cohort of individuals who had sleep problems and were willing to make a change.

### 2.2. Participants and Procedure

**Figure 1:**
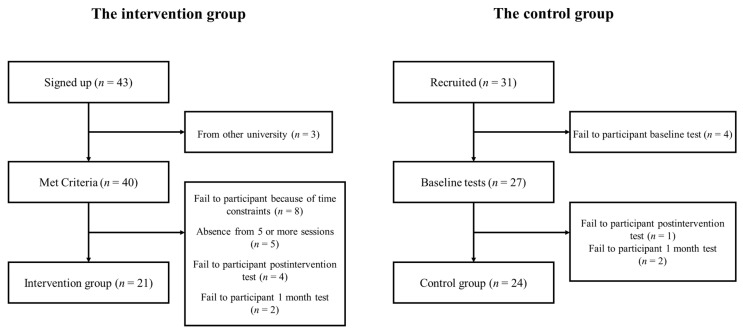
Flow diagram for enrollment.

Students from a university in Beijing who considered themselves to be suffering from sleeping problems were recruited for a mindfulness-based group curriculum through online advertising in WeChat. Advertisements stated, “Have you been in the one of the following situations over a month? (a) Having insomnia at least three days a week; (b) Using your phone more than one hour before you fall into sleep at least three days a week; (c) Having strong and unfavorable emotions including anxiety, tense, depression, frustration, fear, anger, etc. at least three days a week. If you do, you are welcome to our mindfulness-based curriculum. We are looking for individuals over the age of 18 who are not on any kind of pharmacotherapy and have not participated in any kind of mindfulness program”. All the interested students (*n* = 43) completed an online survey in which they provided basic and contact information. Those who met the screening criteria (*n* = 40) were contacted by a research assistant and were provided with additional information about the curriculum and were asked to schedule a baseline assessment (see Figure 1). Of those, eight students failed to participate in the curriculum mainly because of time constraints, and five were excluded from the intervention group due to their absence from five or more sessions. The study was approved by the ethics review committee at the authors’ institute. All participants in the intervention group provided written informed consent for three assessments and eight sessions.

Since most students who were interested but declined to participate for some reason failed to finish the baseline assessment, we had to recruit an unrandomized control group in the current study based on convenience. Students from the corresponding author’s class (*n* = 24) were invited to participate in the three assessments, which included an online survey identical to that completed by the intervention group. All participants in the control group provided written informed consent for three assessments.

Twenty-one university students (14 female; 22.71 ± 4.28 years) with sleep problems were assigned to the intervention group, and twenty-four (19 female; 24.50 ± 0.93 years) students from another class were allocated to the control group. Other demographic information of the participants is presented in Table 1.

### 2.3. Intervention

The group-based MBI in the current study was based on mindfulness-based stress reduction and established group social work techniques. The goal of the intervention was to improve sleep quality and reduce anxiety and depressive symptoms among university students by practicing mindfulness, which means that they would learn to pay attention to what is happening in the present moment with an attitude of acceptance [11,19]. The intervention consisted of eight weekly face-to-face sessions, with each session lasting 120 min. Each session was conducted by either the first author or the corresponding author who had received training and supervision on mindfulness-based stress reduction and group social work. The main mindful contents of eight sessions are described below.

Session 1: This session involved meeting the study participants, expressing expectations and giving information about the concept of mindfulness and the association between mindfulness, stress and sleep. Participants also learned and performed mindfulness-based eating awareness training. In the end, they shared their personal feelings about mindful-eating learning and gave or received feedback to/from group members (See Table 2 for details).

Session 2: In this session, participants performed mindful breathing exercises, which involve a breathing technique to enhance focus and bring awareness to the present moment. Participants, especially those who have difficulty falling asleep, were encouraged to practice mindful breathing every night before they went to bed. They also shared their personal experiences of mindful-eating practicing and mindful breathing learning and gave or received feedback to/from group members.

Session 3: This session entailed a mindfulness body scan, which is a formal mindfulness practice to help increase awareness of bodily sensations, combined with progressive muscle relaxation (PMR), which is a technique where one tense and release all of muscle groups, leaving body feel more relaxed afterward. Participants tended to consider the body scan and PMR as two methods that relieved tension and helped improve sleep quality. In addition, participants shared their personal experiences of mindful learning and training and gave or received feedback to/from group members.

Session 4: In this session, participants performed mindfulness stretching, a mindful practice to enhance focus on body stretching. Mindfulness stretching emphasized the sustained awareness of all body parts and the pleasant or unpleasant feelings brought by any body parts. Participants shared their personal feelings of doing mindfulness stretching and other mindful practicing, as well as the positive effects on sleep of mindfulness practice, and gave or received feedback to/from group members.

Session 5: This session involved reviewing mindfulness stretching and learning Metta meditation, a short mindful practice to increase acceptability of negative thoughts and feelings. Participants were invited to practice Metta meditation in their daily life to help maintain a clam state of mind and stay focused, especially when they were having trouble sleeping. They also shared their feelings during Metta meditation practice and gave or received feedback to/from group members.

Session 6: In this session, participants performed mindful meditation, a mindful practice to enhance focus and bring awareness to the present moment that helps individuals increase the acceptability of thoughts and feelings. Participants were encouraged to do daily exercises of mindful meditation, especially when they have trouble staying focused or falling asleep. In addition, they shared their feelings during mindful meditation and gave or received feedback to/from group members.

Session 7: This session included mindful walking, a mindful movement practice to help increase awareness of daily activities. Participants were encouraged to practice such informal techniques in their daily life and develop a mindful lifestyle, which helps them to find a unique way to deal with their own stress and sleep problems. They also shared their personal experiences of mindful learning and training and gave or received feedback to/from group members.

Session 8: In this session, participants evaluated and summarized all sessions by sharing and reviewing their learning and practicing experience of bringing mindfulness into their daily lives. Participants were invited to continue practicing mindfulness after the intervention finished. In the end, participants expressed and shared their personal feelings towards the group, and each other, and gave or received feedback to/from group members.

### 2.4. Measures

*Sleep outcomes*. Sleep-related outcomes were measured subjectively with the Pittsburgh Sleep Quality Index (PSQI), a scale assessing individuals’ sleep disturbances during the past month [33]. The PSQI, which includes 19 self-rated items and five other-rated items, comprises seven components indicating multiple aspects of sleep situation: *subjective sleep quality*, *sleep latency*, *sleep duration*, *habitual sleep efficiency*, *sleep disturbances*, *use of medication* and *daytime dysfunction*. Each component is scored on a scale of 0–3, and the total PSQI score (0–21) was derived from the sum of the seven component scores. Lower total PSQI scores indicated better sleep quality. In the current study, we only used the 19 self-rated items (e.g., *During the past month, how would you rate your sleep quality overall?*) excluding the five items that needed to be rated by a roommate. The Chinese version of the PSQI has shown good reliability and validity [34]. In the current sample, the Cronbach’s α coefficients of the PSQI and each component ranged from 0.666 to 0.839 across all three assessments.

*Mindfulness*. A Five Facet Mindfulness Questionnaire (FFMQ) was developed to assess the mindfulness of individuals [35]. The FFMQ consisted of 39 items (e.g., *When I’m walking, I deliberately notice the sensations of my body moving*.) and five subscales that tap into the multiple aspects of mindfulness: *observing*, *describing*, *acting with awareness*, *non-judging of inner experience*, and *non-reactivity to inner experience*. Participants responded to the items based on a five-point Likert-type scale ranging from 1 (*never or very rarely true*) to 5 (*very often or always true*), with higher scores indicating a higher level of mindfulness. The Chinese version of the FFMQ has been validated and utilized extensively in nonclinical student samples for the assessment of mindfulness [36]. In the current sample, the Cronbach’s α coefficients of the FFMQ, and each subscale ranged from 0.664 to 0.893 across all three assessments.

*Anxiety symptoms*. Anxiety symptoms was measured using the Self-rating Anxiety Scale (SAS), a 20-item self-report scale describing the frequency of anxiety symptoms (e.g., *I feel more nervous and anxious than usual; I feel afraid for no reason at all*) [37]. Each item was rated on a 4-point Likert scale, ranging from 1 (*none or a little of the time*) to 4 (*most or all of the time*). Five of the items needed to be reversed scored, and higher total scores indicated greater anxiety symptoms. The internal consistency of SAS for the current sample was satisfactory (Cronbach’s α coefficients ranged from 0.739 to 0.890 across all three assessments).

*Depressive symptoms*. Depressive symptoms were assessed using the brief form of the Center for Epidemiological Studies Depression Scale (CES-D), which is a self-report scale consisting of 10 items. The CES-D measured individuals’ depressive symptoms during the last week (e.g., *I feel sad; I have trouble concentrating*). Items were rated on a 4-point Likert scale ranging from 1 (*not at all*) to 4 (*most of the time*). Higher total scores indicated higher levels of depressive symptoms. The Chinese version of the CES-D demonstrated good reliability and validity in student samples [38]. It was also found that the CES-D had good internal consistency (with Cronbach’s α coefficients ranging from 0.863 to 0.895 across all three assessments) in the current sample.

*Demographic characteristics*. Demographic information was obtained during the baseline assessment using an online self-report survey that collected data on each participant’s sex (1 = *male*, 2 = *female*), age, and academic major.

### 2.5. Statistical Analyses

The data were analyzed by the program SPSS version 21.0 (IBM Corp., Armonk, NY, USA). Differences between groups in sociodemographic data and other measures at baseline were evaluated with independent *t*-tests and chi square tests. Variables that were significantly different between groups were treated as covariates in subsequent analyses of intervention effects. Mixed factor analyses of variance were used to test the difference in sleep outcomes (PSQI results) and other mental health symptoms (SAS and CES-D results) between the two groups before, immediately after, and one month after the intervention.

## 3. Results

### 3.1. Descriptive Statistics of the Two Groups at Baseline

The descriptive statistics of the outcome variables at baseline by group are presented in Table 1. There were no significant differences at baseline when comparing the two groups in the subscales of *habitual sleep efficiency* (*t* = 1.00, *p* = 0.327), *sleep disturbances* (*t* = 1.71, *p* = 0.09) *use of medication* (*t* = −0.36, *p* = 0.722) from PSQI, and in the subscales of *observing* (*t* = 0.31, *p* = 0.755), *non-judging of inner experience* (*t* = −1.02, *p* = 0.312), *non-reactivity to inner experience* (*t* = −1.91, *p* = 0.06) from FFMQ. However, the ANOVA results revealed that there were significantly higher levels of PSQI total scores (*t* = 3.47, *p* = 0.001) and other subscales, FFMQ total scores and other subscales (*t* = −3.23, *p* = 0.002), CES-D scores (*t* = 4.96, *p* < 0.001) and SAS scores (*t* = 3.91, *p* < 0.001) in the participants from the intervention group than in the participants from the control group. Those variables that were significantly different between participants in the groups at baseline were considered covariates in the analyses of treatment effects.

### 3.2. Difference in Sleep Outcomes between the Two Groups before and after the Intervention

**Figure 2:**
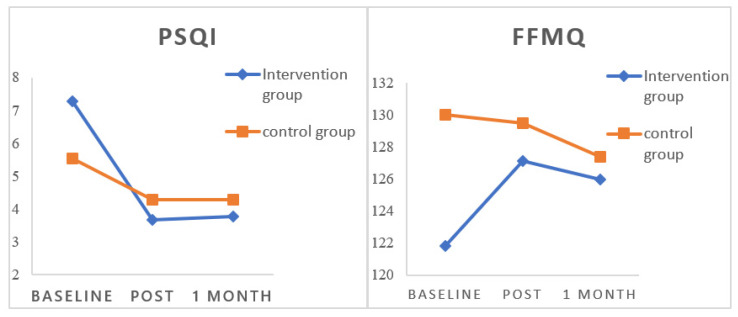
Overall PSQI and FFMQ scores in the two groups over time. Note. PSQI: Pittsburgh Sleep Quality Index; FFMQ: The Five Facet Mindfulness Questionnaire; *n* = 45.

Descriptive and inferential statistics for all sleep-related outcomes in participants in the two groups are presented in Table 3 and Figure 2. The differences in the total PSQI score and seven subscales between the intervention group and control group across the three waves were analyzed using mixed-factor ANOVA. There was a significant interaction effect of group and time on total PSQI score (*F* = 6.94, *p* = 0.012, *η^2^* = 0.15) and the subscale *daytime dysfunction* (*F* = 4.59, *p* = 0.038, *η^2^* = 0.10), although no significant main effect of group or time on total PSQI score (*F_group_* = 0.10, *p* = 0.750, *η^2^* = 0.00; *F_time_* = 1.34, *p* = 0.254, *η^2^* = 0.03) or the subscale *daytime dysfunction* (*F_group_* = 0.22, *p* = 0.641, *η^2^* = 0.01; *F_time_* = 1.08, *p* = 0.304, *η^2^* = 0.03) was found.

### 3.3. Difference in Mindfulness Level between the Two Groups before and after the Intervention

Descriptive and inferential statistics for all aspects of mindfulness in the two groups are presented in Table 4 and Figure 2. Mixed-factor ANOVA was used to analyze the differences in total FFMQ score and the five subscales between the intervention group and control group across the three waves. The results reveal a significant interaction effect of group and time on total FFMQ score (*F* = 4.21, *p* = 0.047, *η^2^* = 0.10) and the subscale of *non-reactivity to inner experience* (*F* = 7.48, *p* = 0.009, *η^2^* = 0.16), although no significant main effect of group or time effect on total FFMQ score (*F_group_* = 0.58, *p* = 0.450, *η^2^* = 0.01; *F_time_* = 0.00, *p* = 0.955, *η^2^* = 0.00) or the subscale *non-reactivity to inner experience* (*F_group_* = 0.01, *p* = 0.911, *η^2^* = 0.00; *F_time_* = 0.43, *p* = 0.518, *η^2^* = 0.01) was found.

### 3.4. Difference in Anxiety and Depressive Symptoms between the Two Groups before and after the Intervention

Descriptive and inferential statistics for anxiety and depressive symptoms in the two groups are depicted in Table 5. The mixed-factor ANOVA revealed that there was neither a significant interaction effect of group and time on anxiety (*F* = 0.28, *p* = 0.600, *η^2^* = 0.01) and depressive symptoms (*F* = 2.03, *p* = 0.162, *η^2^* = 0.05) nor any main effect of group or time on anxiety (*F_group_* = 1.53, *p* = 0.223, *η^2^* = 0.04; *F_time_* = 0.32, *p* = 0.574, *η^2^* = 0.01) and depressive symptoms (*F_group_* = 1.40, *p* = 0.244, *η^2^* = 0.03; *F_time_* = 0.51, *p* = 0.481, *η^2^* = 0.01).

## 4. Discussion

This quasi-experimental study developed and tested the efficacy of a group-based MBI in reducing sleep problems and enhancing mental health among university students. The findings demonstrated that an eight-week group-based MBI improved sleep quality and enhanced mindfulness in university students but had no significant effect on reducing their anxiety and depressive symptoms.

University students’ sleep quality was significantly improved in participants from the intervention group after they completed the eight weekly mindfulness sessions. This result was consistent with previous studies that found sleep could be improved through mindfulness training in other samples [8,15,26,27,28], at least in the short term [32]. From the subscales of the PSQI, we learned that the intervention in the current study had a more significant effect on improving sleep in aspects of daytime dysfunction and overall sleep status than in the other aspects (e.g., sleep latency, habitual sleep efficiency, etc.), which is consistent with several studies that showed the MBI was ineffective for sleep latency or sleep efficiency [4]. In addition, the effectiveness of the intervention was achieved partly because we recruited ‘at-risk’ individuals who considered themselves to have sleep problems and were more likely to be motivated and be ready for a change [39]. Furthermore, it was effective and convenient for university students to practice mindfulness skills in their daily life after learning and mastering such techniques. The group-based MBI also provided an opportunity to share their learning experience and feelings with group members, which reinforced their motivation and achievement [21].

The effect of MBI on improving sleep was reinforced by the findings of the treatment effect of enhancing mindfulness among university students, which were measured by the FFMQ. The students in the intervention group exhibited a significant increase in self-reported mindfulness after the eight sessions, indicating that the intervention enhanced participants’ awareness of mindfulness, which was in line with some previous MBI studies [15,16,40]. Since mindfulness has been found to be closely associated with sleep quality [14], the effectiveness of the intervention for sleep problems which was found in the current study, is probably linked to an increase in mindfulness. Given that a prior study found that benefits of mindfulness training pertaining to sleep quality depended on continued practice [41], the fact that no significant difference in mindfulness of the intervention group between postintervention and 1-month follow-up was probably because of the lack of daily mindful practice among participants after the intervention was finished.

The lack of a treatment effect on anxiety and depressive symptoms in the current study following the MBI may be due to the intervention approach. This outcome probably occurred because we only targeted mindfulness training and sleep sharing during the intervention instead of working on participants’ emotional symptoms. Given the theoretical model linking mindfulness to sleep [24], in which mindfulness cultivates nonjudgmental awareness and creates a calming effect on cognition and physiologic arousal, anxiety and depressive symptoms may not play a part in the mechanism by which mindfulness improve sleep. Another possible reason was that improvement in psychological distress and mental health tends to be a long-term process [20], while the relatively short period of follow-up testing in the present study was not long enough to identify the variance of emotional symptoms.

This study had several limitations that should be considered. First, although the university where participants were recruited is large and comprehensive, the intervention in this study was conducted within only one university, and participants were recruited only through the internet, both of which might exclude part of the population and limit the external generalizability of the findings. Future work should be conducted on a larger sample from multiple universities to provide further evidence regarding the effectiveness of group-based MBI in alleviating sleeping problems among university students. Second, due to the limited number of recruited participants, this study had to adopt a nonrandomized, nonequivalent comparison group, which to some extent introduced bias and restrained the identification of the causal relationships between the intervention and the outcomes. Future studies with randomized controlled trials are anticipated. Third, improvement in sleep was assessed through a self-report approach, but it would be more accurate to measure sleep status through physiological equipment, such as electronic bracelets. Future studies are needed that would include the use of highly accurate physiological indicators. Fourth, the participants in the current study have various kinds of sleeping problems, which led to the heterogeneity of the intervention group. Future interventions for participants with more homogeneous sleeping problems are needed to shed more light on the elaboration and effectiveness of group-based MBI on specific sleeping problems. Finally, this study only provided a starting point for further research regarding the effectiveness of group-based MBI in alleviating sleep problems among university students. Further research could explore the extent to which university students and teachers utilize the mindfulness-based groups available and the mechanism by which MBI reduces sleep problems among students.

## 5. Conclusions

This study employed a group-based MBI to help university students with sleep problems. The intervention in the current study encouraged students with sleep difficulties to focus on the present moment through learning and practicing mindful skills and sharing their experiences with group members in weekly meetings. After eight weekly sessions of mindfulness learning, practicing and sharing, the students in the intervention group enhanced their mindfulness and experienced improved sleep but not reduced anxiety and depressive symptoms. Consequently, the current study contributes to a better understanding of the effectiveness of group-based MBI in addressing sleep problems in university students.

## Figures and Tables

**Table 1:** Demographic characteristics of the two groups at baseline.

Demographic Characteristics	Intervention Group (*n* = 21)			Control Group (*n* = 24)			*t*	*p*
	*M*	*SD*	*n* (%)	*M*	*SD*	*n* (%)		
Age	22.71	4.28		24.50	0.93		−1.874	0.075
Female			14 (66.67%)			19 (79.17%)		
Major categories								
Faculty of Architecture, Civil and Transportation Engineering			5 (23.81%)			0 (0%)		
Faculty of Environment and Life Sciences			5 (23.81%)			0 (0%)		
Faculty of Humanities and Social Sciences			3 (14.29%)			24 (100%)		
Faculty of Information			3 (14.29%)			0 (0%)		
Faculty of Economics and Management			2 (9.52%)			0 (0%)		
Faculty of Science			1 (4.76%)			0 (0%)		
Faculty of Materials and Manufacturing			1 (4.76%)			0 (0%)		
Faculty of Art and Design			1 (4.76%)			0 (0%)		
PSQI	7.86	1.88		5.04	3.28		3.467	0.001
Subjective sleep quality	1.52	0.51		0.83	0.57		4.274	0.000
Sleep latency	1.76	0.83		1.00	0.89		2.965	0.005
Sleep duration	1.00	0.45		0.54	0.72		2.518	0.013
Habitual sleep efficiency	0.43	0.51		0.25	0.68		0.991	0.327
Sleep disturbances	1.14	0.36		0.92	0.50		1.712	0.094
Use of medication	0.05	0.22		0.08	0.41		−0.358	0.722
Daytime dysfunction	1.95	0.81		1.42	0.88		2.119	0.040
FFMQ	118.57	18.04		132.88	11.27		−3.233	0.002
Observing	24.86	6.65		24.25	6.32		0.314	0.755
Describing	24.76	6.79		29.92	4.55		−3.026	0.004
Acting with awareness	25.19	6.36		30.83	5.35		−3.234	0.002
Non-judging of inner experience	24.52	5.35		26.33	6.37		−1.023	0.312
Non-reactivity to inner experience	19.24	4.68		21.54	3.38		−1.911	0.063
CES-D	10.71	4.62		4.42	3.90		4.961	0.000
SAS	35.19	5.95		27.75	6.71		3.910	0.000

Note. PSQI: Pittsburgh Sleep Quality Index; FFMQ: The Five Facet Mindfulness Questionnaire; *n* = 45.

**Table 2:** The content of session 1 of the group-based MBI.

No.	Time	Content
1	10 min	Members self-introduce and get to know each other
2	20 min	Giving information about the concept of mindfulness and association between mindfulness, stress and sleep.
3	40 min	Performing mindfulness-based eating awareness training: mindfully eat raisins
4	30 min	Members sharing their experiences and feelings during the practice of mindfully eating raisins.
5	20 min	Reviewing and sharing the experience and feelings of this session, expressing expectations for the group and the intervention.

**Table 3:** Descriptive and inferential statistics for sleep related outcomes.

	Baseline		Post		1 Month		Group × Time	
	*M*	*SD*	*M*	*SD*	*M*	*SD*	*F*	*η^2^*
Subjective sleep quality							0.76 (ns)	0.02
Intervention group (*n* = 21)	1.52	0.51	1.05	0.67	1.00	0.55		
Control group (*n* = 24)	0.83	0.57	0.50	0.51	0.46	0.51		
Sleep latency							3.05 (ns)	0.07
Intervention group	1.76	0.83	0.95	0.59	1.10	0.63		
Control group	1.00	0.89	0.83	0.64	0.58	0.72		
Sleep duration							1.82 (ns)	0.04
Intervention group	1.00	0.45	0.10	0.30	0.24	0.44		
Control group	0.54	0.72	0.17	0.38	0.21	0.51		
Habitual sleep efficiency							0.28 (ns)	0.01
Intervention group	0.43	0.51	0.05	0.22	0.05	0.22		
Control group	0.25	0.68	0.17	0.38	0.25	0.53		
Sleep disturbances							0.04 (ns)	0.00
Intervention group	1.14	0.36	1.10	0.30	1.10	0.30		
Control group	0.92	0.50	0.83	0.57	0.67	0.57		
Use of medication							5.07 *	0.11
Intervention group	0.05	0.22	0.05	0.22	0.05	0.22		
Control group	0.08	0.41	0.13	0.61	0.21	0.72		
Daytime dysfunction							4.59 *	0.10
Intervention group	1.95	0.81	1.29	0.78	1.29	1.01		
Control group	1.42	0.88	0.87	0.99	1.00	0.93		
Total PSQI Score							6.94 *	0.15
Intervention group	7.86	1.88	4.57	1.54	4.81	1.78		
Control group	5.04	3.28	3.50	2.40	3.38	2.76		

Note. PSQI: Pittsburgh Sleep Quality Index; *n* = 45; ns: *p* > 0.05; *: *p* < 0.05.

**Table 4:** Descriptive and inferential statistics for all aspects of mindfulness (*n* = 45).

	Baseline		Post		1 Month		Group × Time	
	*M*	*SD*	*M*	*SD*	*M*	*SD*	*F*	*η^2^*
Observing							0.62 (ns)	0.02
Intervention group (*n* = 21)	24.86	6.65	25.90	4.76	25.14	6.44		
Control group (*n* = 24)	24.25	6.32	24.29	5.65	23.67	5.48		
Describing							1.48 (ns)	0.04
Intervention group	24.76	6.79	25.48	5.98	25.48	6.19		
Control group	29.92	4.55	29.58	3.93	28.88	4.11		
Acting with awareness							0.12 (ns)	0.00
Intervention group	25.19	6.36	25.10	5.81	25.38	5.61		
Control group	30.83	5.35	30.33	6.45	31.12	5.71		
Non-judging of inner experience							0.63 (ns)	0.02
Intervention group	24.52	5.35	25.10	6.35	24.43	7.00		
Control group	26.33	6.37	26.50	5.67	26.46	5.04		
Non-reactivity to inner experience							7.48 **	0.16
Intervention group	19.24	4.68	22.29	4.97	21.71	5.97		
Control group	21.54	3.38	21.67	4.07	20.58	3.92		
Total FFMQ Score							4.21 *	0.10
Intervention group	118.57	18.04	123.86	18.05	122.14	19.16		
Control group	132.88	11.27	132.37	13.44	130.71	12.13		

Note. FFMQ: The Five Facet Mindfulness Questionnaire; *n* = 45; ns: *p* > 0.05; *: *p* < 0.05, **: *p* < 0.01.

**Table 5:** Descriptive and inferential statistics for anxiety and depressive symptoms.

	Baseline		Post		1 Month		Group × Time	
	*M*	*SD*	*M*	*SD*	*M*	*SD*	*F*	*η^2^*
Anxiety symptoms							0.28 (ns)	0.01
Intervention group (*n* = 21)	35.19	5.95	39.46	9.76	42.44	10.36		
Control group (*n* = 24)	27.75	6.71	38.80	7.53	36.25	9.94		
Depressive symptoms							2.03 (ns)	0.05
Intervention group	10.71	4.62	8.86	4.62	9.29	4.73		
Control group	4.42	3.90	5.17	4.95	5.67	5.48		

Note. *n* = 45; ns: *p* > 0.05.

## Data Availability

All data included in this study are available upon request by contact with the corresponding author.
